# Increased SPHK1 and HAS2 Expressions Correlate to Poor Prognosis in Pancreatic Cancer

**DOI:** 10.1155/2021/8861766

**Published:** 2021-01-06

**Authors:** Mengsi Yu, Kainan Zhang, Song Wang, Li Xue, Zhaoyun Chen, Ning Feng, Conghua Ning, Lijuan Wang, Jing Li, Boke Zhang, Changcheng Yang, Zhaoxia Zhang

**Affiliations:** ^1^Department of Clinical Laboratory, The First Affiliated Hospital of Xinjiang Medical University, Urumqi, China; ^2^Department of Ophthalmology, General Hospital of Xinjiang Military Region, Urumqi, China; ^3^Department of Internal Medicine, 958 Hospital of People's Liberation Army, Chongqing, China; ^4^Clinical Laboratory Center, The First Affiliated Hospital of Anhui University of Chinese Medicine, Hefei, China; ^5^Department of Medical Oncology, The First Affiliated Hospital of Hainan Medical University, Haikou, China

## Abstract

**Objective:**

SPHK1 and HAS2 have been reported to play important roles in tumorigenesis and development. However, their expression and prognostic value in pancreatic cancer (PC) remain unclear. This study is aimed at investigating the expression of SPHK1 and HAS2 on the prognosis of pancreatic cancer.

**Materials and Methods:**

The expression of SPHK1 and HAS2 in pancreatic cancer tissues was analyzed through TCGA and GTEx databases and validated by qRT-PCR and Western blot in pancreatic cancer cell lines. *χ*^2^ test was used to explore the correlation of the SPHK1 and HAS2 expressions with clinical characteristics. Kaplan-Meier survival analysis and ROC curve were used to evaluate the prognostic and diagnostic roles of SPHK1 and HAS2 in pancreatic cancer. Additionally, Spearman correlation analysis was applied to assess the correlation between the SPHK1 and HAS2 in pancreatic cancer. GO analysis and KEGG analysis were applied to explore the possible signaling pathway that SPHK1 and HAS2 coregulated genes mediated.

**Results:**

The expression of SPHK1 and HAS2 was markedly upregulated in pancreatic cancer tissue and cell lines, respectively. Furthermore, there was a significant positive correlation between SPHK1 and HAS2 expressions. ROC curves showed that SPHK1 combine with HAS2 has good diagnostic value in pancreatic cancer patients with 85% sensitivity and 99.4% specificity. Kaplan-Meier analysis showed that increased expression of SPHK1 and HAS2 was significantly associated with short overall survival (OS) of pancreatic cancer patients. GO and KEGG results revealed that SPHK1 and HAS2 mainly involved cell proliferation and invasion mediated by extracellular matrix- (ECM-) receptor interaction, focal adhesion, and PI3K-AKT signaling pathways.

**Conclusions:**

Overexpression of SPHK1 and HAS2 could be important markers for the prognosis of pancreatic cancer.

## 1. Introduction

Pancreatic cancer is one of the most aggressive malignancies. In China, the incidence of pancreatic cancer has increased 6-fold in the past decades. The median survival time of pancreatic cancer patients is only 3-6 months, with a 5-year survival rate of less than 5% [1, 2]. Nowadays, pancreatic cancer remains a poor prognostic cancer type and surgery is the only chance for cure. Pancreatic cancer is characterized by high invasiveness and early metastasis, lacking of specific symptoms in early stage [3]. Only a few patients are able to be cured surgically, due to the late diagnosis. Unfortunately, there is no sensitive and specific biomarker to diagnose pancreatic cancer and predict the prognosis [4].

Sphingosine kinase 1 (SPHK1), key member of the sphingosine kinase (SPHK) family, converts sphingosine to sphingosine 1-phosphate. Sphingosine 1-phosphate enhances cell growth and survival by binding to its receptors. Evidences showed that SPHK1 regulated multiple cellular processes including inhibition of cell apoptosis, promoting cell proliferation, and angiogenesis [5, 6]. Previous studies have suggested that SPHK1 played an important role in tumorigenesis and cancer progression [7]. Recently, SPHK1 expression has been demonstrated to be upregulated in numerous cancers, such as breast cancer, colon cancer, and lung cancer. In these malignancies, SPHK1 commonly functions as an oncogene which promotes cancer metastasis and invasion [8, 9]. However, the expression and clinical significance of SPHK1 in pancreatic cancer remain unknown.

Hyaluronan synthases 2 (HAS2), a member of hyaluronan synthesis family, forms and synthesizes of hyaluronan. Hyaluronan, together with its cell surface receptors, has been shown to contribute to cancer development such as cell migration and invasion [10, 11]. HAS2 has been reported to be correlated with the cancer grade and survival of breast cancer patients [12]. However, there is no study on the association between HAS2 expression and the progression of pancreatic cancer. Our previous work showed that sphingosine 1-phosphate receptor and hyaluronan receptor cooperate to play an important role in cancer lymph-angiogenesis and lymphatic permeability [13, 14]. These discoveries raised the interesting possibility that SPHK1 and HAS2 might play important roles in tumor progression.

The present study examined the expression of SPHK1 and HAS2 in pancreatic cancer tissues and cell lines, respectively. Then, we investigated the correlation of the SPHK1 and HAS2 expressions with clinical characteristic in pancreatic cancer. The possible correlation between SPHK1 and HAS2 expressions in pancreatic cancer was also analyzed. Furthermore, the potential prognostic roles of SPHK1 and HAS2 in pancreatic cancer patients were evaluated. Additionally, we analyzed the protein-protein interaction network of SPHK1 and HAS2.

## 2. Materials and Methods

### 2.1. Data Collection

The Genotype-Tissue Expression (GTEx) database and The Cancer Genome Atlas (TCGA): https://www.cancer.gov/tcga were used to analyze the gene expression profiles of SPHK1 and HAS2 [15]. Gene Expression Profiling Interactive Analysis (GEPIA) database was applied to analyze the expression of SPHK1 and HAS2 in the pancreatic cancer cohort (*n* = 177) and healthy control (*n* = 173). After screening out the tumor types to be studied in our study, the expression of SPHK1 and HAS2 in different stages and the influence of them on the total survival time were further analyzed, in order to explore the roles of SPHK1 and HAS2 in pancreatic cancer. The Human Protein Atlas (http://www.proteinatlas.org) database was used to detect the tissue expression and tissue location of SPHK1 and HAS2 [16].

### 2.2. Cell Culture and Antibodies

Human pancreatic cancer cell line cells (AsPC-1, PANC-1, and Capan-1) were obtained from the ATCC (American Type Culture Collection, Manassas, USA). Normal pancreatic duct epithelial HDPE6C7 cells were obtained from Type Culture Collection Cell Bank (Chinese Academy of Sciences Committee, Shanghai, China). Cells were cultured in Dulbecco's Modified Eagle Medium (Gibco BRL, Grand Island, NY, USA) supplemented with 10% fetal bovine serum in a humidified atmosphere of 5% CO_2_. Rabbit polyclonal anti-SPHK1 and rabbit anti-HAS2 were purchased from Santa Cruz Biotechnology (Santa Cruz, CA, USA).

### 2.3. qRT-PCR

TRIzol Reagent (Invitrogen) was used to extract the total RNAs. Spectrophotometer (NanoDrop ND-2000, Thermo Fisher Scientific, Waltham, MA, USA) was applied to determine the purity and concentration of RNAs. We first used cDNA synthesis kits (MaiGene, Binhai, Tianjin) according to the protocols in the kits. SYBR Green RT-PCR was performed to measure mRNA levels, which were then calculated by using the 2^-*ΔΔ*Ct^ method. Primers were as follows: SPHK1, 5′-CATTATGCTGGCTATGAGCAG-3′ (forward) and 5′-GTCCACATCAGCAATGAAGC-3′ (reverse). HAS2, 5′-TGAACAAAACAGTTGCCCTTT-3′ (forward) and 5′-TTCCCATCTATGACCATGACAA-3′ (reverse).

### 2.4. Western Blot

Cells were lysed in 1% Triton X-100 lysis buffer. The total protein concentration in the cell lysate was determined using a BCA protein assay kit (Pierce, Rockford, IL). Proteins from each sample were separated on 10% SDS-PAGE and transferred to polyvinylidene fluoride membrane (PVDF). The PVDF membranes were blocked with Tris-buffered saline (TBS) containing 5% nonfat milk powder at 37°C for 2 h, and immunoblot analysis was performed with mouse anti-GAPDH, rabbit anti-SPHK1, and rabbit anti-HAS2 at 4°C for 12 h. The membranes were washed with TBS/T buffer for three times and then incubated with HRP-conjugated polyclonal secondary antibody for 1 h at 37°C. The membranes were developed with the enhanced plus chemiluminescence assay (Pierce) according to the manufacturer's instructions. Images were analyzed using the Image-Pro Plus 6.0 software.

### 2.5. Correlation between SPHK1 and HAS2 Expressions

In order to investigate the correlation of SPHK1 and HAS2, Spearman correlation analysis was applied to research the relationship of SPHK1 and HAS2. To investigate the diagnostic values of SPHK1 and HAS2, ROC curve was used to assess to calculate the AUC, specificity, and sensitivity of each biomarker. In addition, ROC curve was used to assess the combined diagnostic efficacy of SPHK1 and HAS2. The prognostic effects of SPHK1 and HAS2 for overall survival (OS) of pancreatic cancer patients were evaluated by Kaplan-Meier survival analysis.

### 2.6. GO Function Analysis

The genes coregulated by SPHK1 and HAS2 were sorted out, which were identified by correlation coefficient > 0.6 or *P* < 0.01. In addition, Cluster Profile was used to perform Gene Ontology (GO) functional enrichment analysis for genes [17].

### 2.7. Statistical Analysis

All statistical analyses were performed by using the SPSS 22.0 software (IBM SPSS, Chicago, IL) and the R software 3.6.1 (R Foundation for Statistical Computing, Vienna, Austria). The association between SPHK1 and HAS2 expressions in pancreatic cancer was analyzed using Spearman's correlation. *χ*^2^ tests were used to detect the correlation of the SPHK1 and HAS2 expressions with clinical characteristics. Overall survival (OS) was analyzed by Kaplan-Meier survival curves with 95% confidence intervals (CIs), and the differences between subgroups were compared by log-rank test. *P* < 0.05 was considered statistically significant.

## 3. Results

### 3.1. The Expression of SPHK1 and HAS2 Was Significantly Upregulated in PC

**Figure 1:**
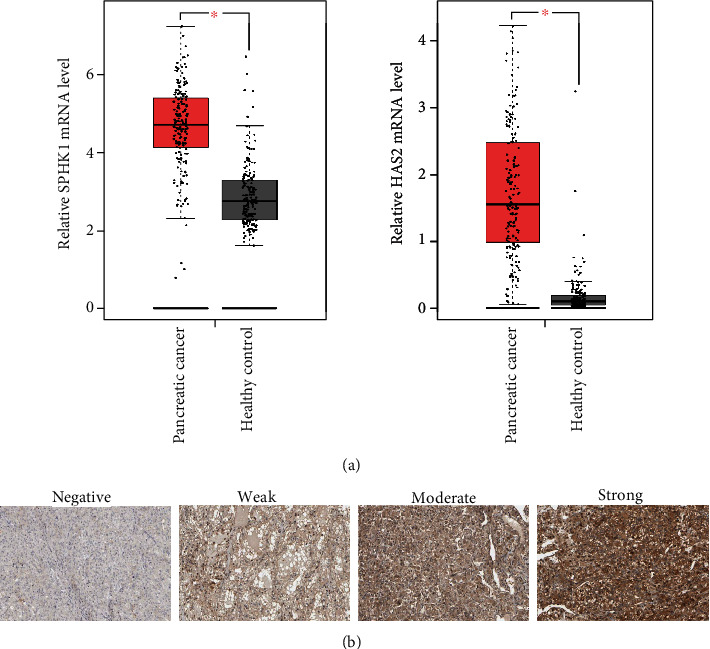
SPHK1 and HAS2 are upregulated in pancreatic cancer. (a) Relative SPHK1 and HAS2 mRNA expressions in pancreatic cancer patient tissues versus normal samples from TCGA and GTEx databases. (b) Immunohistochemical staining of SPHK1 in normal pancreatic and pancreatic cancer tissues on the Human Protein Atlas database. The “weak,” “moderate,” and “strong” correspond to the low, medium, and high of the intensity of immunohistochemical staining; ^∗^*P* < 0.05.

To explore the potential roles of SPHK1 and HAS2 in pancreatic cancer, TCGA and GTEx databases were used to analyze the expression of SPHK1 and HAS2. The results showed that the mRNA expression of SPHK1 and HAS2 was significantly higher in pancreatic cancer group (177 cases) than those of the healthy control (173 cases) (*P* < 0.05 and *P* < 0.05), respectively (Figure 1(a)). We observed the protein expression of SPHK1 in pancreatic cancer and normal pancreatic tissues using immunohistochemical staining on the Human Protein Atlas. The representative images indicated that SPHK1 protein expression was strongly enhanced in metastatic pancreatic cancer tissues compared with normal pancreatic tissue (Figure 1(b)). SPHK1 was mainly localized in the cytoplasm of cancer cells, and there was no SPHK1 expression in normal pancreatic tissue.

### 3.2. The Expression of SPHK1 and HAS2 Was Increased in PC Cell Lines

**Figure 2:**
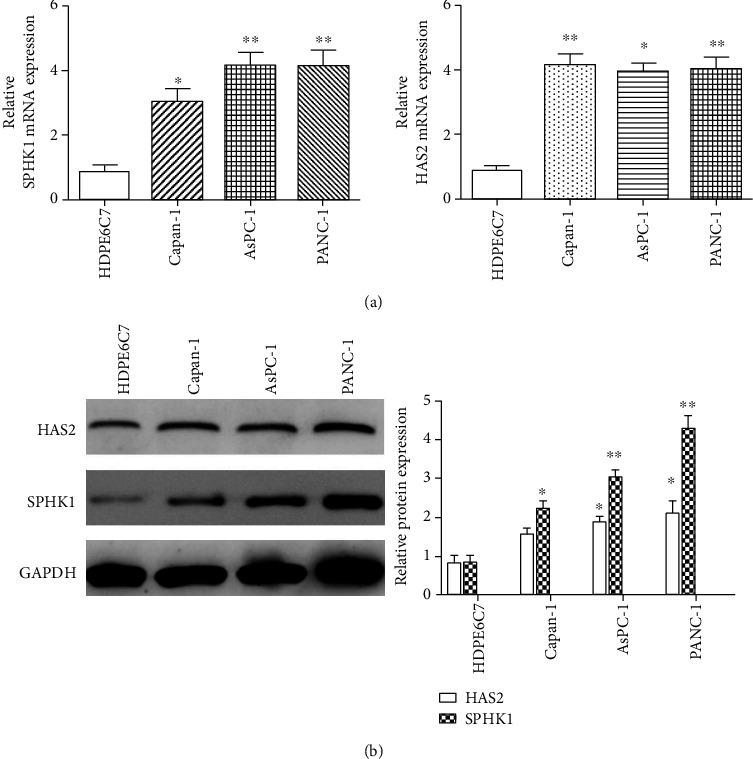
Overexpression of SPHK1 and HAS2 in pancreatic cell lines. (a) qRT-PCR was used to detect the expression of SPHK1 and HAS2 mRNA expressions in pancreatic cell lines (Capan-1, AsPC-1, and PANC-1) and normal pancreatic duct epithelial HDPE6C7 cell line. (b) Western blot analysis of SPHK1 and HAS2 expressions in pancreatic cancer and cell lines (AsPC-1, Capan-1, and PANC-1) and normal pancreatic duct epithelial HDPE6C7 cells. ^∗∗^*P* < 0.01 and ^∗^*P* < 0.05.

To further verify the expression of SPHK1 and HAS2 in pancreatic cancer, we detected the mRNA and protein expressions of SPHK1 and HAS2 in pancreatic cancer cell lines. Consistent with the findings from the pancreatic cancer tissues, the result of qRT-PCR showed that the mRNA expression of SPHK1 and HAS2 was increased in pancreatic cancer cell lines (Capan-1, AsPC-1, and PANC-1), in comparison to normal pancreatic duct epithelial HDPE6C7 cell line, respectively (*P* < 0.05, *P* < 0.01, and *P* < 0.01) (Figure 2(a)). The protein expression of SPHK1 and HAS2 was validated by Western blot, and the results showed that the levels of SPHK1 and HAS2 protein were markedly elevated in pancreatic cancer cell lines compared with normal pancreatic duct epithelial cell line (Figure 2(b)).

### 3.3. Correlation of the SPHK1 and HAS2 Expressions with Clinical Characteristics

To further explore the roles of SPHK1 and HAS2 in pancreatic cancer, a preliminary analysis was performed to identify whether the expression of SPHK1 and HAS2 in pancreatic cancer tissues was associated with clinicopathological parameters. According to the median values of SPHK1 and HAS2 expressions, pancreatic cancer patients were divided into high and low SPHK1 and HAS2 expression groups. *χ*^2^ tests revealed the relationship between the SPHK1, HAS2 expression, and the clinical characteristics. As shown in Table 1, no significant association was found between HAS2 expression and all the clinical features. However, the result demonstrated that pancreatic cancer patients with high SPHK1 expression were significantly related to the location of tumor in the pancreas (*P* = 0.007), clinical stage (*P* = 0.013), and tumor status (stage T, *P* = 0.03).

### 3.4. The Correlation and Diagnostic Values of SPHK1 and HAS2 in PC

**Figure 3:**
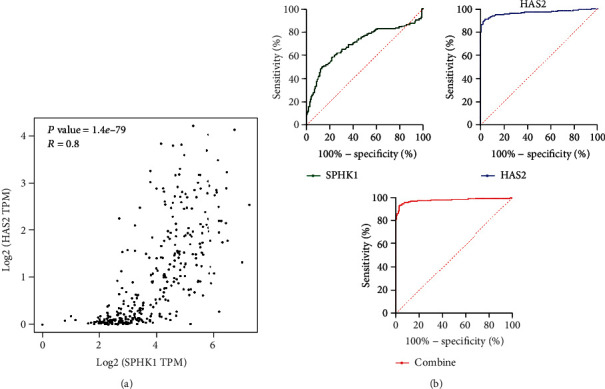
The positive correlation and diagnostic ROC curves of SPHK1 and HAS2 in pancreatic cancer. (a) Correlation between SPHK1 and HAS2 expressions in pancreatic cancer tissues analyzed by TCGA and GTEx databases. (b) Diagnostic ROC curves of SPHK1, HAS2, and SPHK1 combine with HAS2 in distinguishing pancreatic cancer and normal people in a TCGA cohort.

Based on these above data that revealed the expression of both SPHK1 and HAS2 in pancreatic cancer was upregulated, we analyzed the correlation of SPHK1 and HAS2 in pancreatic cancer. TCGA database showed that there was a strong positive correlation (*P* < 0.001 and *R* = 0.8) between SPHK1 and HAS2 expressions (Figure 3(a)). The possible potential application of SPHK1 and HAS2 in distinguishing pancreatic cancer was further explored. The diagnostic ROC analysis of SPHK1 and HAS2 in TCGA cohort showed that SPHK1 and HAS2 can serve as potential diagnostic biomarkers for pancreatic cancer (*P* < 0.05). As shown in Figure 3(b), SPHK1 [AUC (95%CI) = 0.74 (0.669‐0.792)] exhibited a high diagnostic value with 70% sensitivity and 98.4% specificity (P <0.001). HAS2 [AUC (95% CI) =0.989 (0.969-0.997)] exhibited a high diagnostic value with 80% sensitivity and 99.4% specificity (*P* < 0.001). Notably, SPHK1 combine with HAS2 has better diagnostic value with 85% sensitivity and 99.4% specificity (*P* < 0.001).

### 3.5. SPHK1 and HAS2 Both Predict a Poor Prognosis for PC Patients

**Figure 4:**
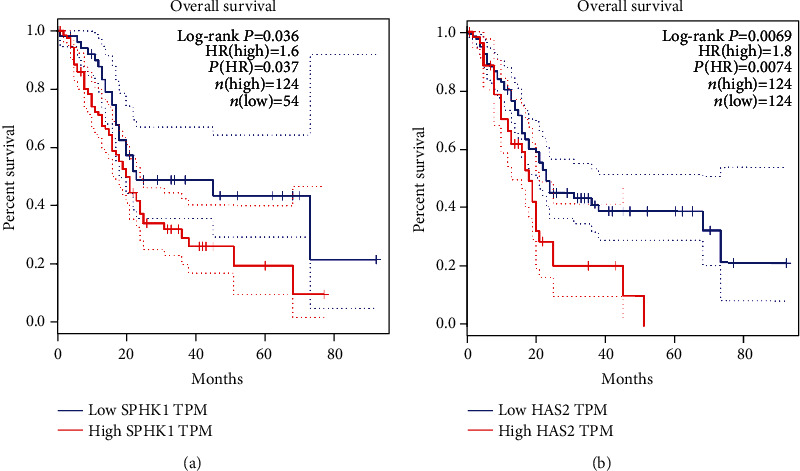
The prognostic significance of SPHK1 and HAS2 in pancreatic cancer patients. (a) Kaplan-Meier curves showing the OS of pancreatic cancer patients with high and low SPHK1 expression. (b) Kaplan-Meier curves showing the OS of pancreatic cancer patients with high and low HAS2 expression. ^∗^*P* < 0.05.

The prognostic effects of SPHK1 and HAS2 for overall survival (OS) of pancreatic cancer patients were evaluated by Kaplan-Meier survival analysis and the log-rank tests. The results revealed that pancreatic cancer patients with high expression of SPHK1 had shorter OS than patients with low SPHK1 expression (*P* = 0.037) (Figure 4(a)), and pancreatic cancer patients with high HAS2 expression had shorter OS than patients with low expression of HAS2 (*P* = 0.0074) (Figure 4(b)).

### 3.6. Bioinformatics Analysis of SPHK1 and HAS2 Coregulated Genes

**Figure 5:**
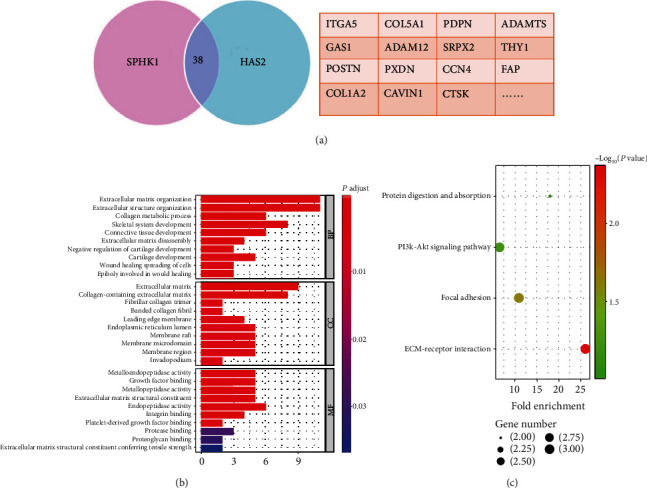
GO term and KEGG analysis of the coregulated genes. (a) 38 genes were coregulated by SPHK1 and HAS2. (b) GO term enrichment. *Y*-axis shows the GO terms of biological process. The length of the bars is proportional to the number of genes. (c) KEGG enrichment. The size of the nodes is proportional to the number of genes. GO: Gene Ontology; KEGG: Kyoto Encyclopedia of Genes and Genomes.

There were 38 coregulated genes predicted to be target genes of SPHK1 and HAS2. The genes were listed in the form (Figure 5(a)). In order to further explore the biological function of SPHK1 and HAS2, GO and KEGG analyses were applied to predict the possible signaling pathway which coregulated genes mediated. GO term enrichment analysis of coregulated genes revealed that the biological processes mainly involved in extracellular matrix (ECM) organization and spreading of cells (Figure 5(b)). KEGG pathway analysis showed that coregulated genes mainly involved in ECM-receptor interaction, focal adhesion, and PI3K-AKT signaling pathways (Figure 5(c)). All this signal pathways were verified to participate in multiple cell biological functions, including cell metabolism, apoptosis, and migration. The results of GO and KEGG analyses suggested that SPHK1 and HAS2 might play important roles in pancreatic cancer cell proliferation and invasion.

## 4. Discussion

Pancreatic cancer is difficult to diagnose in its early stages, and fewer than 20% of patients are eligible for curative resections [18]. The prognostic factors are indispensable for providing individualized treatment of pancreatic cancer patients [19]. Carbohydrate antigen 19-9 (CA19-9), the only authenticated marker for clinical application, is deficient in the specificity. The majority of other markers are expensive or experimental and are not widely used in routine clinical practice [20]. Therefore, there is an urgent need to identify novel sensitive and specific biomarkers for improving the diagnosis and accurately evaluating the prognosis of pancreatic cancer. In this study, we analyzed the expression and clinical significance of SPHK1 and HAS2 in pancreatic cancer.

SPHK1 and HAS2 have previously been demonstrated to be upregulated in several types of human cancer and to play an important role in tumor development and progression, including cellular motility and cellular proliferation and angiogenesis [21, 22]. Previous studies have reported that the expression of SPHK1 and HAS2 was elevated in a variety of malignant tumor tissues [23, 24]. Therefore, we analyzed TCGA and GTEx databases to evaluate the expression of SPHK1 and HAS2 in pancreatic cancer. The results showed that SPHK1 and HAS2 expressions were increased in pancreatic cancer tissues compared with normal tissues. This result is consistent with the previous founding. Consistently, the expression of SPHK1 and HAS2 in pancreatic cancer cell lines was significantly increased. Notably, in the present study, three pancreatic cancer cell lines were selected for evaluating the SPHK1 and HAS2 expressions. Capan-1 and AsPC-1 were derived from metastatic site, while PANC-1 was epithelioid origin. Our data (Figure 2) showed that the epithelioid-derived PANC-1 expressed more SPHK1 and HAS2, compared to these metastatic site-derived Capan-1 and AsPC1, suggesting that SPHK1 and HAS2 may be related to the origin of pancreatic cancer cell lines. The increased expression of SPHK1 and HAS2 in pancreatic cancer implied that SPHK1 and HAS2 may be essential for the progression of pancreatic cancer. Moreover, high SPHK1 expression is significantly related to tissue location, clinical stage, and tumor status. Previous study showed that SPHK1 may exert metastatic and invasive effects by upregulation of CD44 (hyaluronan receptor) expression through the ERK signaling pathway in colon cancer cells [25]. Additionally, our previous work showed that sphingosine 1-phosphate receptor and hyaluronan receptor cooperate to play an important role in cancer lymph-angiogenesis and lymphatic permeability, suggesting that the receptor of SPHK1 product directly interacts with the receptor of HAS2 product [13, 14]. HAS2 is well known in various tumor invasions [26], so the interaction of SPHK1 and HAS2 might exert a pivotal role in pancreatic cancer progression.

Then, Spearman correlation analysis was performed, and the results indicated that there is a significantly positive correlation between the expression of SPHK1 and HAS2 in pancreatic cancer. We have verified that the expression of SPHK1 and HAS2 was markedly increased in pancreatic cancer tumor tissues. Thus, we consider that SPHK1 and HAS2 may be potential biomarkers for pancreatic cancer diagnosis. Undoubtedly, the ROC curves showed that the area under the curves (AUC) of the SPHK1 and HAS2 were both more than 0.7. SPHK1 combined with HAS2 has higher diagnostic efficacy than any single indicator. However, the expression of SPHK1 and HAS2 was more easily detected in the advanced stages of pancreatic cancer. Thus, SPHK1 and HAS2 might not be ideal biomarkers in the detection of early-stage pancreatic cancer. Kaplan-Meier analysis showed that increased expression of SPHK1 and HAS2 was associated with short overall survival (OS) of pancreatic cancer patients. Therefore, it is clinically possible to detect SPHK1 and HAS2 to improve the diagnosis and prognosis of pancreatic cancer. Recent studies have demonstrated the possible potential molecular pathways for SPHK1 and HAS2 in cancer progression, one of which is that SPHK1 induces lung cancer cell migration and paclitaxel resistance by modulating IGF-1-induced EMT [27]. HAS2 mediated hyaluronan production through Notch1 activation and liver fibrosis [28, 29]. The GO and KEGG results showed that the coregulated genes mainly involved in cell invasion, cell adhesion, and positive regulation of angiogenesis by ECM-receptor interaction, focal adhesion, and PI3K-AKT signaling pathways. The common signaling pathway of SPHK1 and HAS2 in pancreatic cancer might be responsible for cancer progression.

In conclusion, this study demonstrated for the first time that the expression of SPHK1 and HAS2 was markedly increased in pancreatic cancer. Additionally, there was a significant correlation between SPHK1 and HAS2. Furthermore, the combination of SPHK1 and HAS2 could be an effective diagnostic marker for the pancreatic cancer. However, it should be noted that SPHK1 and HAS2 might not be sensitive in the early diagnosis of pancreatic cancer. Most importantly, both SPHK1 and HAS2 were significantly associated with short OS of pancreatic cancer patients. Thus, SPHK1 and HAS2 might be useful prognostic biomarkers for pancreatic cancer and worthy of further study before their use in clinical practice.

## Figures and Tables

**Table 1:** Correlations between the expression of SPHK1, HAS2, and clinicopathological characteristics in pancreatic cancer.

Characteristics		Total	HAS2		*P*	SPHK1		*P*
			High	Low		High	Low	
Age	≥49	163	80	83	0.75	81	82	0.28
	<49	14	9	5		8	6	
Gender	Male	97	44	53	0.17	49	48	1.00
	Female	80	45	35		40	40	
Stage	I	21	10	11	0.907	6	15	0.003
	II	146	74	72		78	68	
	III+IV	7	4	3		3	4	
	NA	3	-	-		-	-	
T stage	T1+T2	31	14	17	0.48	10	21	0.03
	T3+T4	144	75	69		78	66	
	NA	2						
N stage	N0	49	21	28	0.98	23	26	0.50
	N1	123	63	60		65	58	
	NA	5	-	-		-	-	
Tumor location	Head of pancreas	129	60	69	0.10	71	58	0.007
	Body of pancreas	15	10	5		6	9	
	Tail of pancreas	14	5	9		3	11	
	Other	19	13	6		8	11	

^∗^ *P* < 0.05.

## Data Availability

Data are available on request.
